# Digital interaction design promotes cultural identification and heritage-related participation intention: Evidence from the Wangchuan ceremony

**DOI:** 10.1371/journal.pone.0362093

**Published:** 2026-11-12

**Authors:** Yun Liu, Kaiting Duan

**Affiliations:** Xiamen Academy of Arts and Design, Fuzhou University, Xiamen, China; Liverpool John Moores University, UNITED KINGDOM OF GREAT BRITAIN AND NORTHERN IRELAND

## Abstract

The sustainable protection of intangible cultural heritage faces unprecedented challenges due to the process of urbanization and the decline in the participation of the younger generation. Digital reconstruction offers a transformative way for heritage transmission, but its long-term contribution to cultural sustainability remains under-explored. This study examines the relationships among users’ perceptions of digital heritage experiences, cultural identification, and heritage-related behavioural intention. On the basis of the Technology Acceptance Model (TAM), the study introduces emotional design and cultural identification into the model and analyzes data from 220 valid questionnaires using partial least squares structural equation modeling (PLS-SEM). The results show significant positive associations between emotional design and perceived enjoyment, between perceived enjoyment and cultural identification, and between cultural identification and heritage-related behavioural intention. Cultural identification shows the strongest association with behavioural intention, suggesting its important role in linking users experiential responses with subsequent heritage-related behavioural intentions.These findings suggest that, if intangible cultural heritage wants to genuinely serve the sustainable development of culture, it should not remain at the level of static documentation, but should strive to create an interactive experience that can trigger emotional resonance, so as to drive the public’s cultural identification. The evaluation framework provided by this study may provide reference for the design and evaluation of similar digital heritage projects.

## Introduction

The United Nations Sustainable Development Goals (SDGs) state that the sustainable protection of intangible cultural heritage is the cornerstone of global social cohesion and cultural diversity [1,2]. However, in the face of rapid urbanization and global cultural homogenization, traditional rituals, which are the vivid embodiment of intangible cultural heritage, are experiencing a profound “sustainability crisis” [3]. Due to the ageing of inheritors and the weakening interest of the younger generation, the material inheritance of these rituals is increasingly hindered.The Wangchuan ceremony (送王船仪式), a maritime ritual jointly inscribed by China and Malaysia on UNESCO’s Representative List of the Intangible Cultural Heritage of Humanity, exemplifies this challenge [4]. Although digital reconstruction has become a transformative tool for heritage documentation, most existing work remains limited to static archiving and fails to foster a deeper sense of cultural identification, which is essential for the long-term transmission of cultural heritage [5]. Therefore, a shift from passive digital preservation to emotion-driven digital transformation is needed to support the long-term transmission of such rituals in the digital media environment.

Current efforts in the digital preservation of intangible cultural heritage have concentrated primarily on “static digitization” — high-fidelity 3D scanning and archival recording of artifacts and ritual settings [6]. While valuable for documentation, such work often ignores the dynamic social transformation required for the continuation of living heritage in contemporary society.Without active public participation and emotional input, digital archives may become “digital tombs” and cannot promote inter-generational knowledge inheritance, which is crucial to cultural sustainable development [7]. The missing link in current digital heritage strategies is a comprehensive understanding of how interactive experience translates into the deep cultural identification that fundamentally motivates communities to safeguard their heritage.

To address this gap, the study proposes a comprehensive evaluation framework based on the Extended Technology Acceptance Model (TAM), which incorporates emotional resonance design and cultural identification as core variables.The Wangchuan ceremony was selected as the case study because, as cross-border cultural heritage shared by communities along the Maritime Silk Road, its survival depends not only on the continuation of the ritual itself but also on whether the collective memory and shared values it carries can be evoked [8,9]. By reconstructing the ritual through interactive digital media, the study hopes to verify whether immersive technology can shift users from passive spectators into active participants in cultural transmission.

The study aims to provide empirical evidence on the relationships among user perceptions of digital heritage experiences, cultural identification, and heritage-related behavioural intention. Using partial least squares structural equation modeling on data from 220 participants, the study examines the hypothesized relationships among perceived design, behavioural design, emotional design, perceived enjoyment, cultural identification, and heritage-related behavioural intention. The findings provide empirical insights into how users experiential perceptions are associated with cultural identification and subsequent intentions to engage with and further explore intangible cultural heritage.

## Methods

### 2.1. Theoretical framework and model development

The Technology Acceptance Model (TAM) proposed by Davis (1989) [10] is one of the most classic and widely used theoretical frameworks in the field of information systems, used to explain and predict users acceptance of information technology.Its core purpose is to understand which factors determine the users adoption and use of new technologies.The Technology Acceptance Model believes that when it comes to the acceptance behaviour of information technology, individuals will produce Perceived Usefulness (PU) and Perceived Ease of Use (PEOU) under the action of external factors., these two variables are the key factors that affect the individual’s Attitude Towards Using (ATU) and Behavioral Intention to use (BI).These four constructs constitute the core elements of the original model. After that, in TAM 3 proposed by Venkatesh and Bala (2008) [11], Perceived Enjoyment (PE) was officially identified as one of the key pre-dependent variables of perceived ease of use (PEOU).Based on the TAM 3 model, the study explores the factors affecting the public’s adoption of different technologies in the process of cultural heritage digital dissemination.The proposed theoretical model is shown in Fig 1.

**Fig 1:**
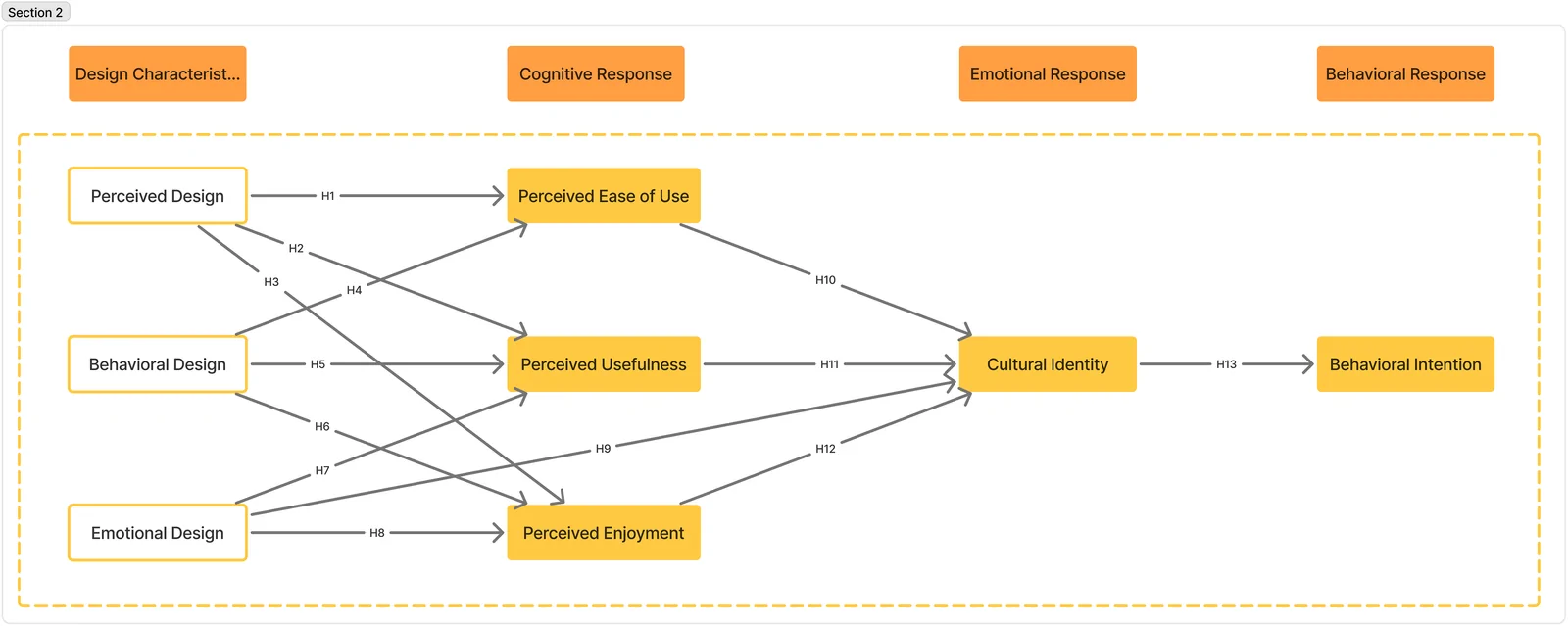
Theoretical model framework. Created by the authors.

#### 2.1.1. Perceptual design (PD).

Perceived design activates user experience through the sensory system. Users exchange information with the external environment through vision, hearing, smell, and touch. Appropriately enhancing users sensory experience can improve their overall experience as well as the communicative effectiveness of installation artwork [12,13]. In intangible heritage ritual contexts, it is necessary to transform the originally complex ritual process into intuitive and visually legible information, and reproduce the cultural situation of the ritual with appropriate light, shadow and multimedia means.Based on the above analysis, this study proposes the following hypotheses:

H1: Perceived design (PD) is positively associated with users perceived ease of use (PEOU).

H2: Perceived design (PD) is positively associated with users Perceived Usefulness (PU).

H3: Perceived design (PD) is positively associated with users Perceived Enjoyment (PE).

#### 2.1.2. Behavioral design (BD).

Behaviour design refers to the dynamic characteristics and operation experience of the interaction process between the user and the device. It focusses on whether the physical action of the body is coordinated with the feedback of the system, specifically showing whether the gesture recognition is accurate, whether the operation is smooth, whether the feedback is timely, and whether the task process is coherent [14,15]. The goal of this design dimension is to transform the actions that rely on the body to complete in the ceremony into an operable digital interactive form. Based on the above analysis, this study proposes the following hypotheses:

H4: Behavioral Design (BD) is positively associated with users perceived ease of use (PEOU).

H5: Behavioral Design (BD) is positively associated with users Perceived Usefulness (PU).

H6: Behavioral Design (BD) is positively associated with users Perceived Enjoyment (PE).

#### 2.1.3. Emotional design (ED).

Emotional Design focuses on creating an appropriate situational atmosphere for users, evoking their intrinsic emotional responses through multimodal narrative techniques [16,17]. It goes beyond functionality and aesthetics, and helps users establish a connection with cultural meaning with emotional resonance. In the digital scene of intangible heritage rituals, Emotional Design plays the role of conveying the emotional core and cultural value of the ritual, transforming users from simple “bystanders” to active “feelers”and thereby reflection and identification.Based on the above analysis, this study proposes the following hypotheses:

H7: Emotional Design (ED) is positively associated with users Perceived Usefulness (PU).

H8: Emotional Design (ED) is positively associated with users Perceived Enjoyment (PE).

H9: Emotional Design (ED) is positively associated with users Cultural Identification (CI).

#### 2.1.4. Perceived ease of use (PEOU).

Perceived ease of use refers to users subjective judgment of the effort required to use the digital installation. If users find the device easy to learn and operate, perceived ease of use is considered high. This constitutes the baseline of user experience.Only when the technological threshold does not constitute an obstacle can users pay more attention to understanding the cultural content itself. Based on the above analysis, this study proposes the following hypotheses:

H10: Users perceived ease of use (PEOU) is positively associated with users Cultural Identification (CI).

#### 2.1.5. Perceived usefulness (PU).

Perceived Usefulness refers to users subjective assessment of the effectiveness of the digital installation in helping them understand and learn relevant knowledge.In the context of intangible cultural heritage rituals, “usefulness” does not refer to task efficiency but rather to cognitive and cultural value; that is, whether the installation helps users understand the ritual process, its symbolic meanings, and its cultural background more effectively and in greater depth than traditional forms of presentation.Based on the above analysis, this study proposes the following hypotheses:

H11: Users Perceived Usefulness (PU) is positively associated with users Cultural Identification (CI).

#### 2.1.6. Perceived enjoyment (PE).

Perceived enjoyment (PE) refers to users subjective sense of enjoyment, interest, and engagement when encountering a digital heritage experience [18]. It represents an intrinsic experiential response that may encourage users to further explore the associated cultural content. Based on the above analysis, this study proposes the following hypotheses:

H12: Users Perceived Enjoyment (PE) is positively associated with users Cultural Identification (CI).

#### 2.1.7. Cultural identification (CI).

Cultural Identification (CI) refers to the sense of belonging, emotional connection, and value identification that an individual or group forms toward a particular culture, manifested as the acceptance and preservation of common history, traditions, beliefs, customs, and symbols [19]. Zhang Dainian [20], in An Introduction to Chinese Culture, argues that the identification of cultural spirit is the foundation of cultural heritage-related participation intention.Within digital ICH communication, cultural identification represents a higher-level psychological response reflecting users sense of belonging, emotional connection, and recognition of cultural value. When users develop stronger identification with the heritage, they may also show greater willingness to engage in heritage-related activities and further explore its cultural meaning. Based on the above analysis, this study proposes the following hypotheses:

H13: Cultural identification (CI) is positively associated with heritage-related behavioural intention (BI).

#### 2.1.8. Behavioral intention (BI).

Behavioral intention (BI) refers to users subjective willingness and perceived likelihood to engage in future behaviors related to the intangible cultural heritage discussed in the experience. It includes intentions to recommend and share the digital heritage experience, revisit similar heritage experiences, and further participate in or explore the related intangible cultural heritage [21]. Under the framework of sustainable development, Behavioral intent is an important indicator for measuring cultural resilience. Digital devices that provides only “ease of use” without stimulating “Behavioral Intention “cannot meaningfully contribute to the heritage ecosystem. Therefore, the important path from Cultural Identification to Behavioral Intention found in the study confirms that digital transformation must give priority to value internalization rather than stop at static digital records.

### 2.2. Digital system implementation

#### 2.2.1. The Wangchuan ceremony.

Intangible cultural heritage rituals in the Fujian–Taiwan region represent the living heritage of the integration of Chinese maritime civilization and regional culture. Their formation and development are rooted in the shared historical trajectory and geographical environment across the Taiwan Strait.The Wangchuan ceremony provides a representative example. Originating in southern Fujian, China, more than 600 years ago, it spread to Taiwan and parts of Southeast Asia through population migration and maritime trade. It has evolved into a marine sacrifice activity with regional characteristics, carrying communities reverence for the sea and aspirations for peace, safety, and prosperity.On 17 December 2020, the Wangchuan ceremony, jointly nominated by China and Malaysia under the title Wangchuan ceremony, Rituals and Related Practices for Maintaining the Sustainable Connection Between Humanity and the Ocean, was inscribed on UNESCO’s Representative List of the Intangible Cultural Heritage of Humanity.The Wangchuan ceremony reflects the concept of harmonious coexistence between humans and nature while reinforcing the sustainable relationship between humanity and the ocean [22].

As a living cultural heritage shared by China and Malaysia, the Wangchuan ceremony serves as an important vehicle for promoting social sustainability.The ritual is sustained through extensive community participation. From the construction of the Wangchuan boat to the ritual procession, the entire process generates substantial social capital and strengthens the ties among communities connected by migration along the Maritime Silk Road.However, in contemporary society, the continuity of this social structure is challenged by two major pressures. First, rapid urbanization and youth out-migration are weakening the social, material, and demographic foundations that sustain traditional ritual practices.However, contemporary Wangchuan practices also involve environmental considerations. The burning of the Wangchuan boat, which forms an important part of the ritual in many communities, involves the combustion of substantial physical materials and may generate atmospheric emissions and material residues. The frequency, scale, and specific practices vary across communities in Fujian, Taiwan, and other regions, so the environmental implications should be understood within their local contexts rather than treated as a uniform or severe environmental problem [23].

In this context, digital reconstruction can serve as a complementary strategy rather than a replacement for living ritual practice. A digital–physical hybrid model may allow selected symbolic and participatory elements to be experienced digitally while, where appropriate, reducing the frequency or scale of physical combustion. Such an approach seeks to balance cultural continuity and environmental responsibility without assuming that digital practice should replace the traditional ceremony.This preserves cross-border cultural connection and social resilience while also mitigating the environmental impact of heritage practice.In this sense, digital transformation also helps align the safeguarding of maritime tradition with the goals of the 2030 Agenda for Sustainable Development concerning cultural preservation, climate action, and marine ecosystem conservation [24]. Field photographs of the ceremony are shown in Fig 2.

**Fig 2:**
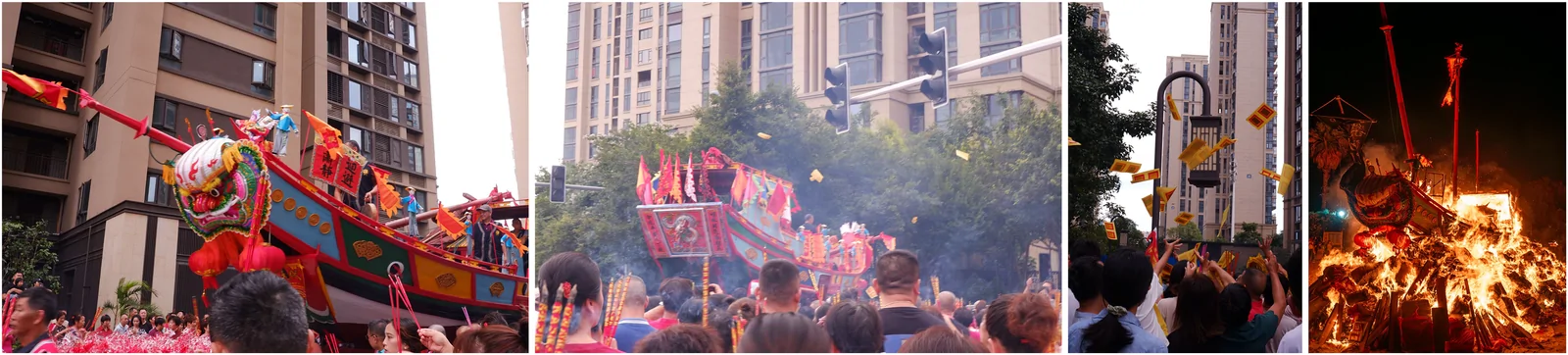
On-site photographs of the Wangchuan ceremony. The photographs document the ritual boat procession through the community (parading the boat through local streets) and the burning of the ritual boat. Photographs taken by the authors.

#### 2.2.2. Ritual symbol translation.

The Wangchuan folk ritual of the Fujian-Taiwan region is a comprehensive cultural display combining artistry, aesthetics, entertainment, and religiosity.To translate this living ritual into a digital interactive experience, the design first identifies and interprets the ritual’s symbolic system. Following Krippendorff’s design semantics [25], a product’s form, color, and structure should transcend physical function to become a symbolic system that conveys meaning, guides interaction, and constructs cognition. The design semantics of the installation were therefore analyzed from three dimensions: cultural, emotional, and functional.

At the cultural level, the design integrates the egg, a universal symbol of life, with the Wangchuan boat, a key ritual symbol, translating the abstract concept of the continuity of life into a tangible ritual form. As a result, the installation itself functions as a medium for cultural expression.At the level of emotional semantics, the sense of sacredness conveyed by white and the sense of warmth conveyed by orange form a contrasting pair of emotional cues, echoing both the solemnity of the ritual and the collective emotion required for community cohesion, allowing users to simultaneously feel the ritual’s sacredness and its human warmth.In terms of functional semantics, the semi-closed interactive field defined by the arc opening, combined with the circular gear symbolizing operation and continuity, establishes a clear interaction narrative，which not only suggests the display and interaction function of the product, but also hints at its role as a carrier of dynamic cultural process (Fig 3).

**Fig 3:**
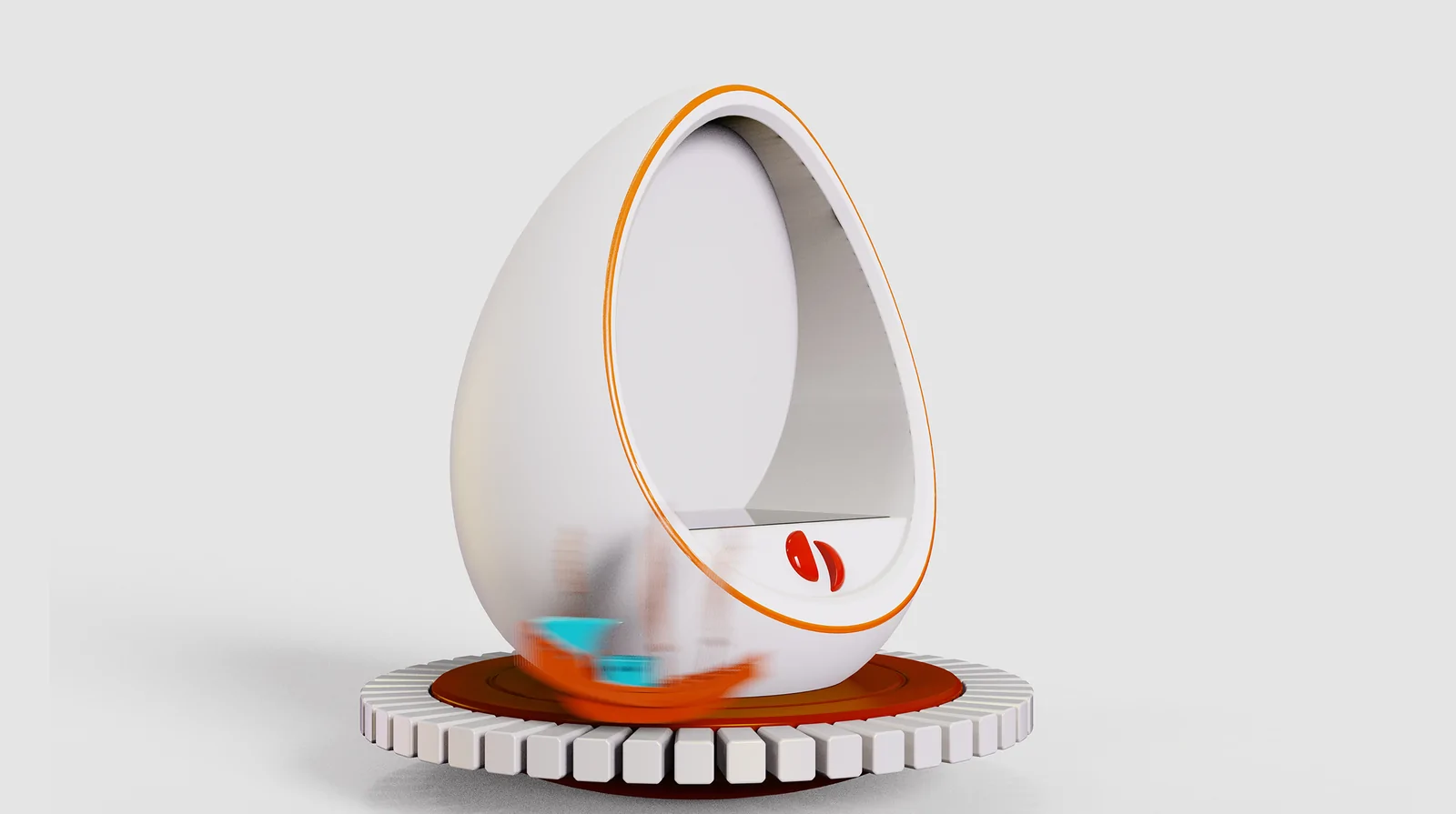
Design of the Wangchuan ceremony Boat Form. Created by the authors.

Jiaobei (筊杯) (divination blocks) serve as a normative ritual symbol in the Wangchuan ceremony, with their sacredness and authority sustained through shared cultural beliefs and ritual conventions. During the ritual, their significance derives not only from their physical wooden form but also from the complete sequence of ritual actions—throwing the blocks, observing how they land, and interpreting the outcome. Three outcomes are possible.During the ceremony, the sanctity of the jiaobei stems not only from their wooden form but, more importantly, from the complete behavioral chain of “casting – landing – interpretation.”A Shengbei (圣杯) (one convex side and one flat side facing upward) indicates the deity’s approval and provides clear guidance. A Xiaobei (笑杯) (two convex sides facing upward) suggests that the request is unclear, the timing is not yet appropriate, or the deity has not reached a decision, requiring the participant to seek guidance again. A Yinbei (阴杯) (two flat sides facing upward) indicates disapproval.As the opening stage of the ritual, this divination process was retained in the digital design, together with the inherent randomness of both the act of throwing the jiaobei and its outcomes (Fig 4).

**Fig 4:**
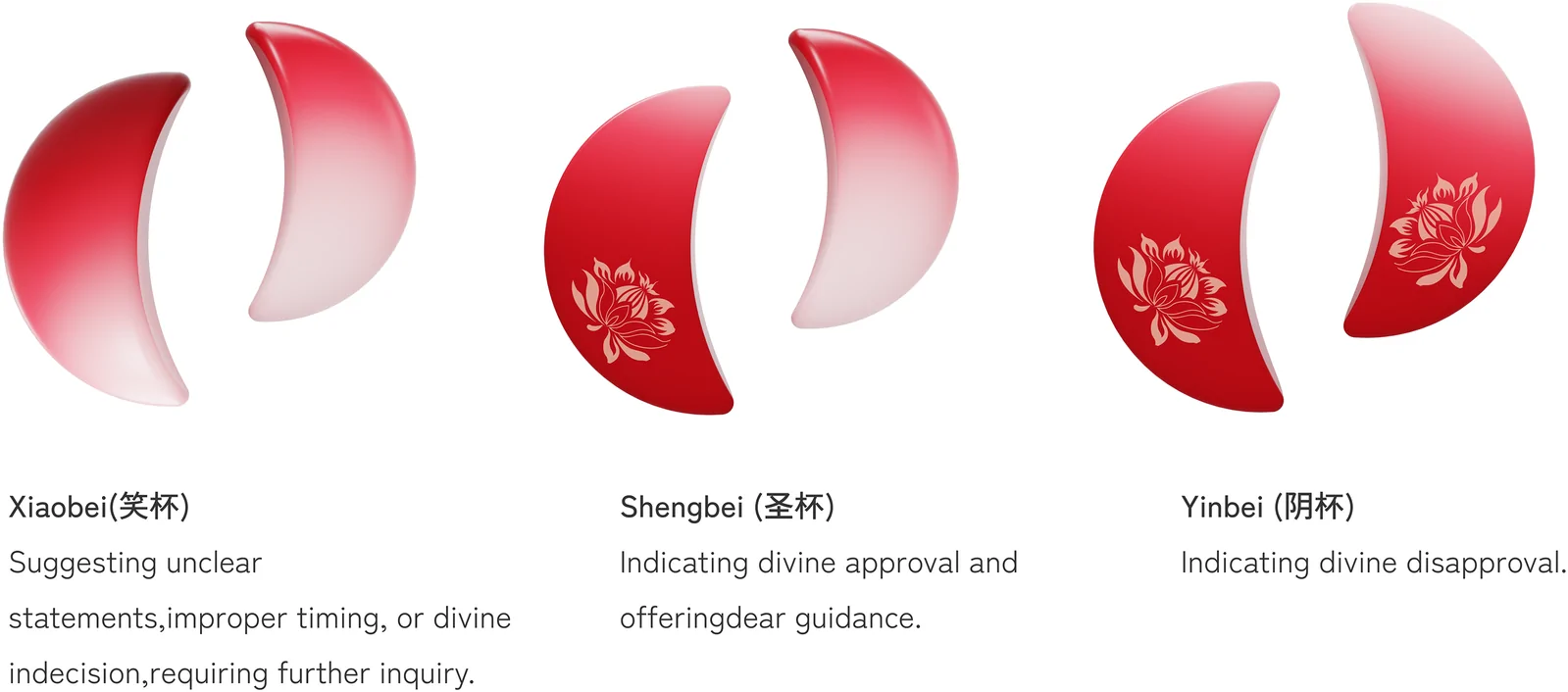
Design of the Jiaobei. Created by the authors.

The deities Wangye (王爷) and Mazu (妈祖) are the two most influential maritime deities of the Fujian-Taiwan region.It should be noted that “Wangye” is not a single deity but a collective term for a group of deities, each with a distinct local origin story.Most were historical figures who contributed to their local communities during their lifetimes and were deified after death. Their functions have continually expanded with evolving social needs, gradually becoming an important symbol of public blessing.As a deity in Chinese folk belief, Wangye does not have a single historical or physical prototype. Rather, the deity’s appearance has been shaped through long-term artistic representation in temple sculptures, wood carvings, paintings, and other forms of folk art. Therefore, this study did not attempt to digitally replicate any specific statue or wood carving. Instead, a collection of representative visual materials depicting Wangye—including temple sculptures, wood carvings, and other publicly available cultural images—was compiled and analyzed. Based on both these visual references and descriptions in the literature, key visual characteristics, including facial features, facial expression, headdress, costume, and color palette, were identified and synthesized into a set of visual design keywords.

These keywords were subsequently used as prompts in JiMeng AI (ByteDance), a diffusion-based image generation platform, to iteratively generate multiple candidate representations of Wangye. The generated images were then reviewed and refined through discussions within the research team. Finally, a preliminary evaluation involving domain experts was conducted to assess the cultural authenticity, visual consistency, and ritual appropriateness of the generated designs. The final image adopted in the interactive system was selected based on consensus among both the research team and the experts (Fig 5).

**Fig 5:**
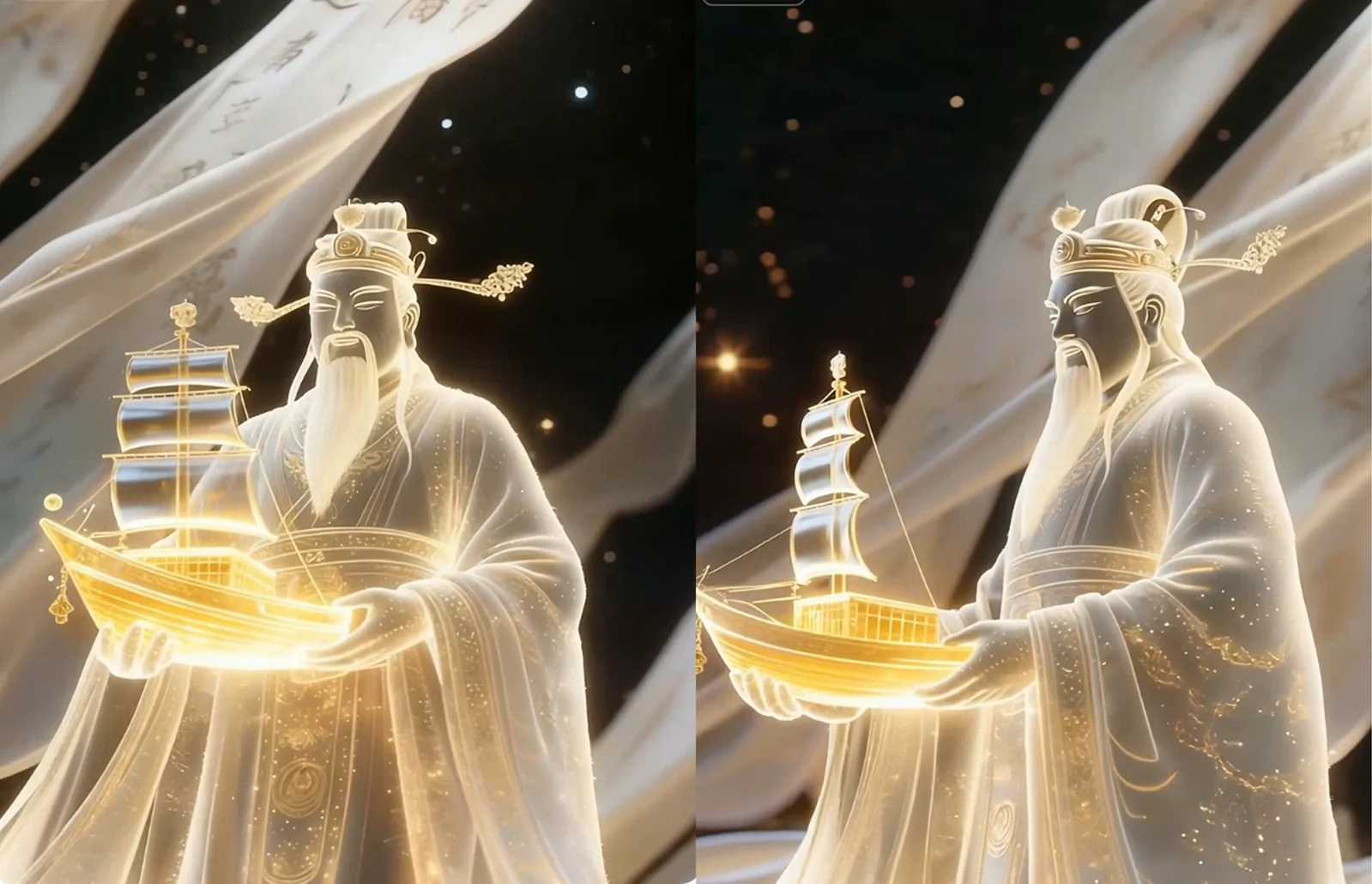
AI-generated Wang Ye imagery. Created by the authors.

#### 2.2.3. Interaction design translation.

To further clarify how digital reconstruction reshapes ritual participation and cultural transmission, this study proposes a framework mapping the relationships among ritual symbols, digital interaction, and design strategy (Fig 6 and S1 Video). This framework conceptualizes the digital transformation of the Wangchuan ceremony as a layered, progressive process, in which ritual symbols are first translated by digital technology into interactive experiences, and then further realized as concrete design strategies that shape how users participate.

**Fig 6:**
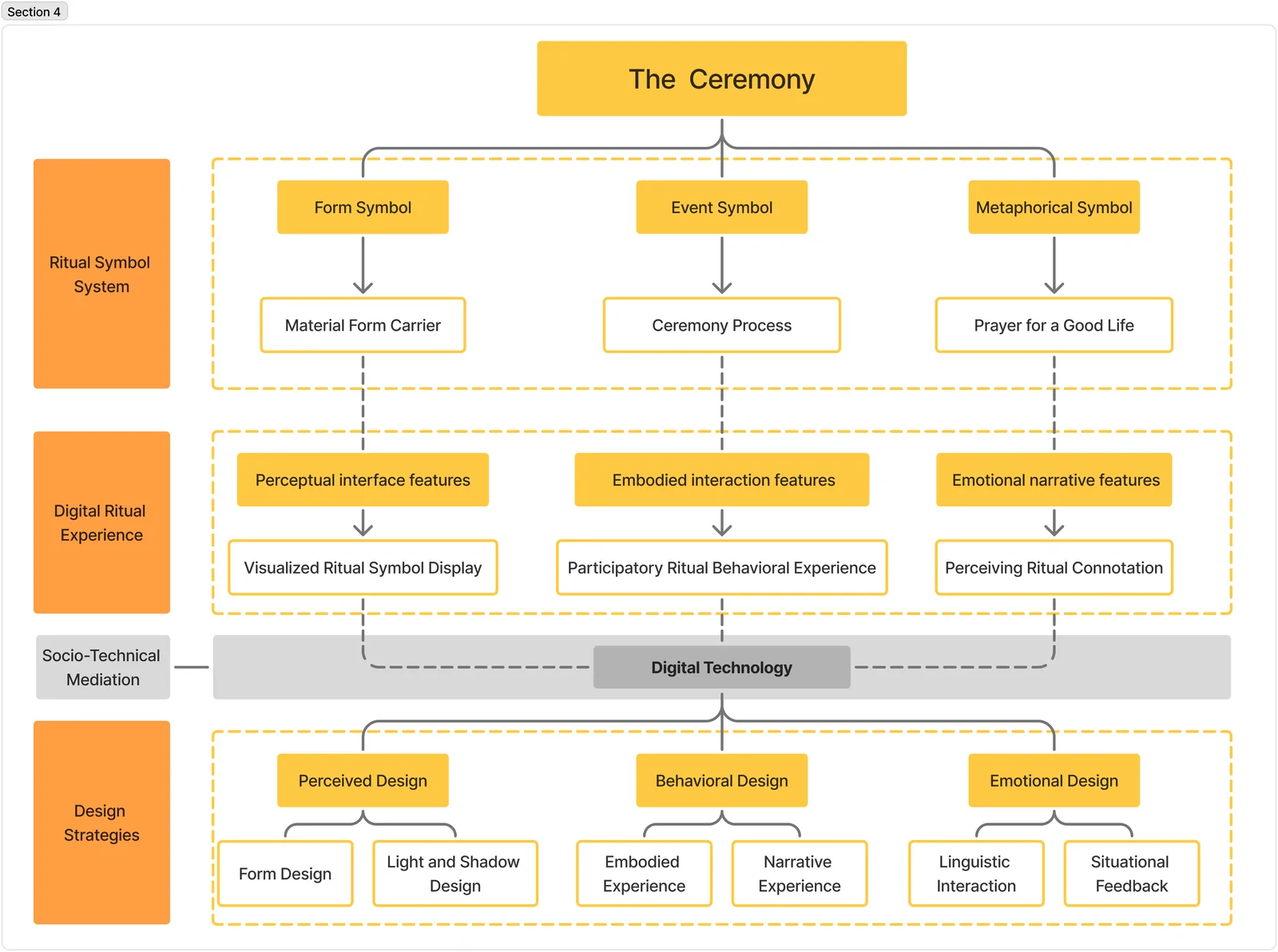
Research framework for interaction design translation of the Wangchuan ceremony. Created by the authors.

At the first level, the framework treats the ritual’s symbolic system as its cultural foundation, comprising three categories: formal symbols, event symbols, and metaphorical symbols. Formal symbols refer to the material carriers of ritual meaning, such as the structure of the Wangchuan boat and related ritual objects (e.g., jiaobei blocks). Event symbols represent the collective ritual process, including the procession, petition, Pudu (普度), and ceremonial performance. Metaphorical symbols express the ritual’s deeper cultural values, such as prayers for peace, prosperity, and community well-being. These three types of symbols together constitute the cultural narrative that digital reconstruction hopes to interpret and convey.

At the second level, these symbolic elements are translated into concrete digital ritual experiences through perceptual interface features, embodied interaction features, and emotional narrative features. The perceptual interface visualizes ritual symbols within the digital environment, allowing users to observe and interpret ritual elements (e.g., the egg-shaped form, light and shadow, and the visual representation of the Wangchuan boat). Embodied-interaction features simulate participatory ritual behavior, enabling users to engage in ritual activity within a virtual environment (e.g., casting jiaobei, guiding the boat). Emotional narrative features further help users understand the meaning of rituals through storytelling and situational cultural narrative (e.g., blessing slips, the burning scene). In this transformation process, digital technology serves as the intermediary connecting “symbolic structure” with “experiential design”: with the help of immersive visualization, interactive interfaces, and narrative systems, the digital platform transforms ritual participation, which was originally limited to a specific place and time, into a more accessible and flexible cultural experience.

At the design output level, the framework identifies three complementary strategies — perceptual design, behavioral design, and emotional design — which together shape how users perceive, experience, and interpret ritual practice within the digital environment. Through these three design strategies, digital reconstruction extends ritual participation beyond the physical community and, to some extent, promotes broader cultural exchange. Overall, this transformation suggests that digital technology serves not only to preserve ritual knowledge but also to reshape modes of participation and interpretation in the transmission of cultural heritage. With the help of new forms of interaction and narrative participation, the digital heritage platform is expected to support the long-term survival of the marine ritual tradition.

The complete Wangchuan ceremony consists of multiple stages, including divination, erecting the ritual lamp pole, constructing the Wangchuan boat, welcoming the deity, worshiping the deity, ritual procession, Pudu, and the ceremonial burning of the Wangchuan boat. Through narrative design, these stages were distilled and reorganized into four core interactive phases: jiaobei divination (掷筊), petition (请愿), Pudu, and sending off the deity (送王).

#### 2.2.4. Emotional resonance strategy.

According to James W. Carey’s ritual view of communication [26], communication is fundamentally a process through which culture is enacted and shared through participation. From this perspective, rituals are not merely the repetition of established practices but a means of creating, maintaining, and transmitting cultural meaning.In the digital era, people’s commemorative rituals have changed markedly: they are no longer confined to the mechanical repetition of past commemorative behaviors, but are instead expressed through the coordinated use of multiple symbols, reflected in a sense of ritual, immersion, and identification.

Digital technology can be used to reconstruct the Wangchuan ceremony and the dynamic dissemination of this ceremony can be realised.The interaction design translates the four core phases into operable digital actions, with clear user freedom, system feedback, cultural semantics, and emotional goals (Fig 7; Table 1).

**Table 1:** User operations, system feedback, and emotional goals in the four interaction stages.

Stage	What users can do	System feedback	Emotional goal
Jiaobei divination (掷筊)	Click/gesture to cast jiaobei; choose whether to cast again	Randomly generates shengbei/xiaobei/yinbei, corresponding to divine response	Establish a human–deity connection and transition from everyday concerns to ritual intention
Petition (请愿)	Input or select a wish; may skip	Displays blessing text/imagery	Introspection and human–divine dialogue
Pudu (普度)	Guide the virtual Wangchuan boat; choose a route	Turbulent or smooth voyage, corresponding to real-world obstacles or positive turning points	Compassion, empathy, and collective care
Sending off (送王)	Watch the boat burning; click to finish; generate a blessing slip	Flames, stardust, and blessing slips	Letting go, hope, and sharing

**Fig 7:**
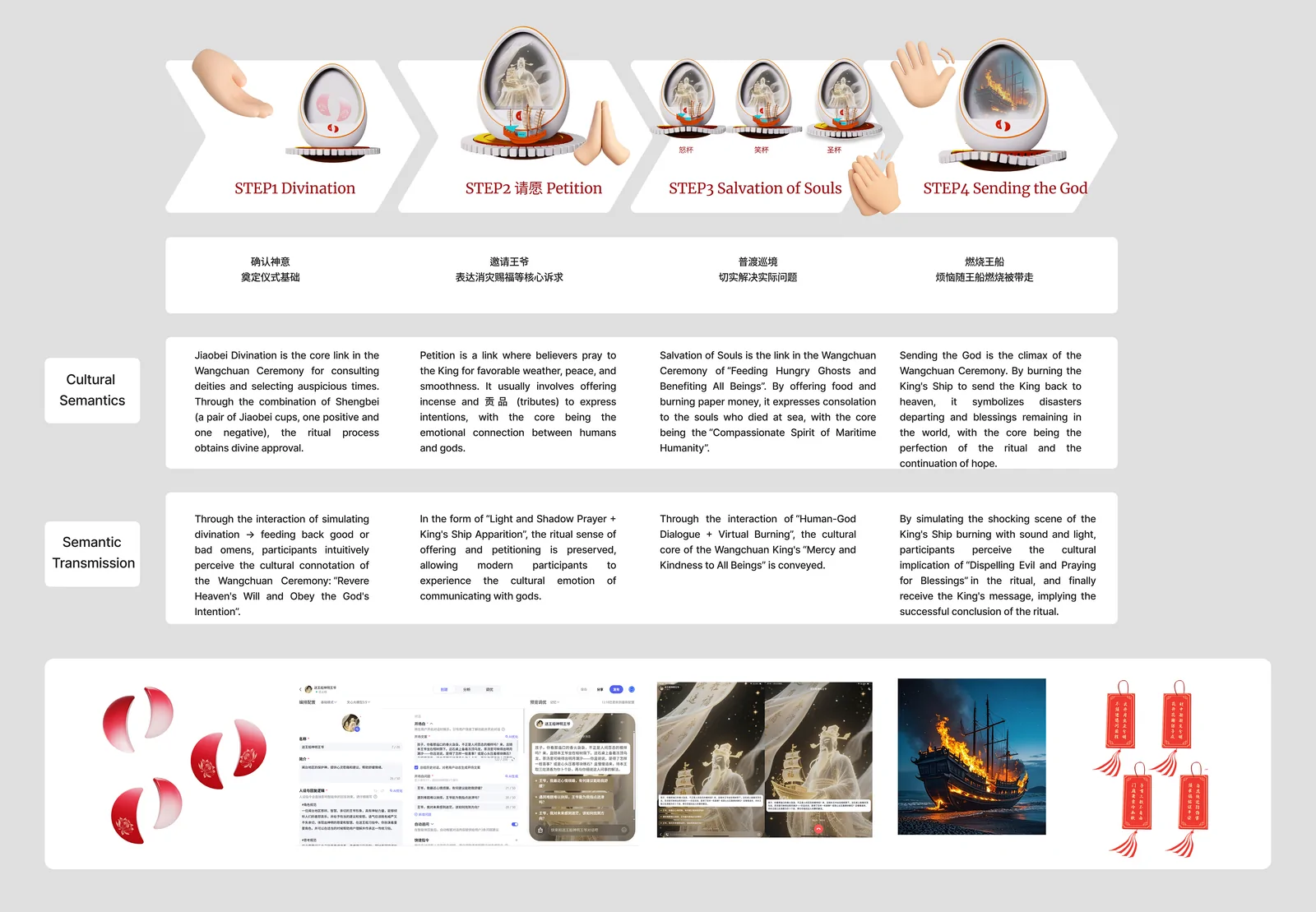
Behavioral interaction flow diagram. Created by the authors.

Jiaobei divination (掷筊). During this phase, the interaction design simulates the ritual act of divination. Users can cast the jiaobei virtually and choose whether to cast again; they cannot control the outcome. The system randomly generates shengbei, xiaobei, or yinbei, each corresponding to a different divine response. This interaction establishes an initial sense of connection between the participant and the deity, facilitating an emotional transition from everyday concerns to ritualized intention.

Petition (请愿). In the petitioning stage, the design focuses on expressing inner emotion. Users can input or select a wish and may skip this step; they cannot make wishes on behalf of others. A “human–divine dialogue” format constructs a private, introspective communicative space, reinforcing the psychological experience of “harmony between human and divine.” The system responds with Blessing text or imagery.

Pudu (普度). During the Pudu phase, the design seeks to convey the deity’s compassion and benevolence while responding to participants’ real-world concerns. Users guide a virtual boat along a chosen route; they cannot alter the fundamental meaning of Pudu. The outcome of the virtual jiaobei divination is then translated into the sailing behaviour of the virtual boat. A turbulent voyage corresponds to real-world obstacles, while a smooth voyage suggests a positive turning point. The design also evokes the ritual meaning of compassion toward wandering spirits by incorporating virtual offerings and blessing slips, linking users personal concerns to the collective moral obligation of care. The system provides visual and auditory feedback corresponding to the boat’s progress.

Sending off the deity (送王). During the concluding phase, the ritual reaches its conclusion, symbolizing the departure of misfortune. After the voyage ends, the dialogue naturally concludes, or the user clicks to finish. Users can watch the burning of the Wangchuan boat and generate a blessing slip; they cannot stop the ending. The scene of the boat burning symbolizes the dissipation of misfortune and the rebirth of hope. The system also generates visual signatures according to the navigation trajectory, transforming abstract encouragement into symbols that can be disseminated and shared. This completes the transformation from a virtual ritual experience to real-world psychological support. The ritual completion and blessing slip design is shown in Fig 8.

**Fig 8:**
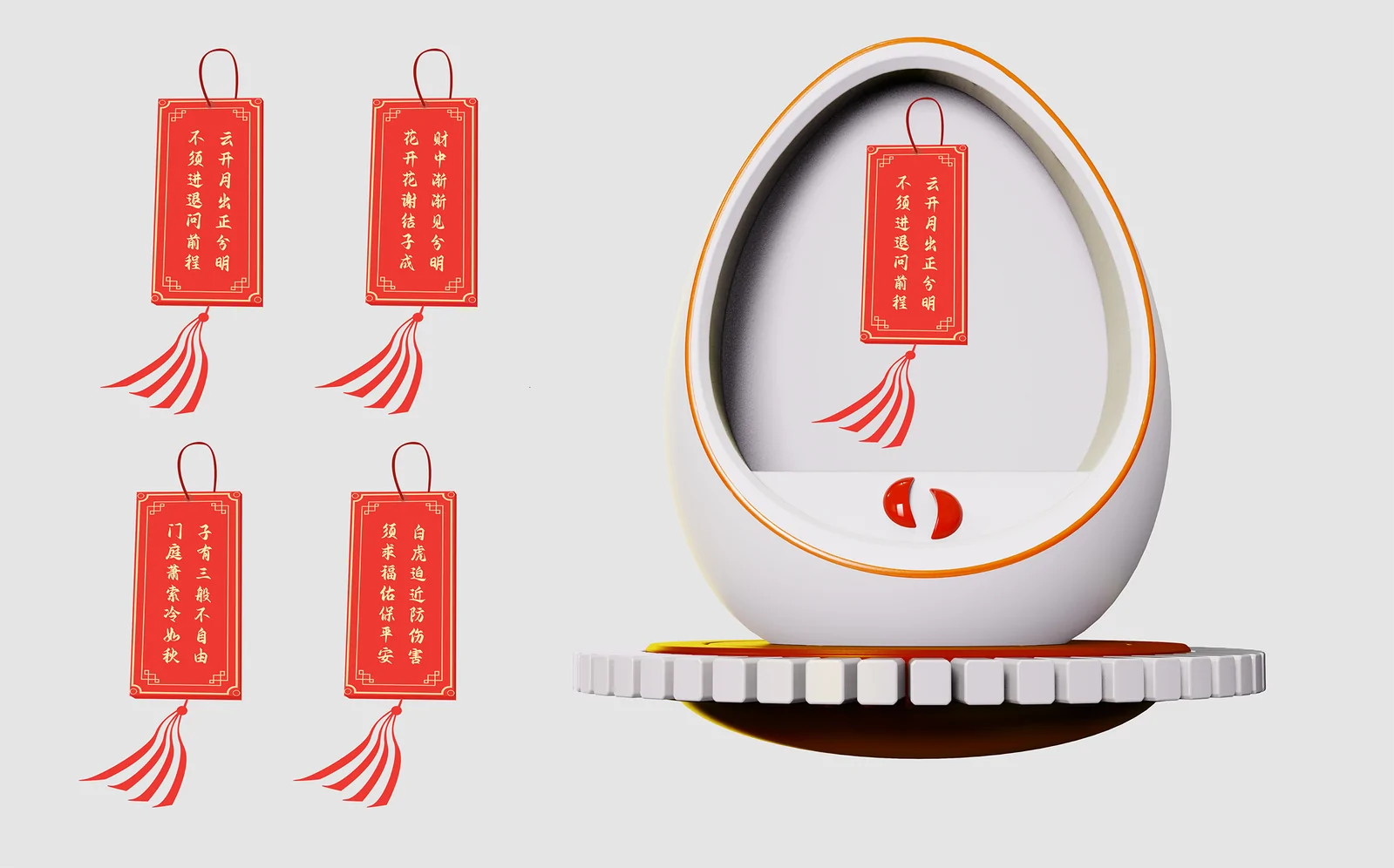
Ritual completion and blessing slip. Created by the authors.

In the digital era, people express personal psychological needs from a first-person perspective through posting, sharing, and commenting online, using blessing images, videos, and other digital media to voice their wishes [27]. The design practice uses the ritual as the main thread to construct the product’s user flow, reinforcing embodied ritual experience.Using the complete Wangchuan ceremony sequence as the guide for structuring interaction steps, users are no longer mere observers; through embodied operations, they become deeply immersed in the situational and spiritual core created by the ritual, gaining an experience that combines bodily perception with cultural resonance.At the same time, the design also relies on the emotional clue of “good wishes” to deepen the cultural narrative connotation of the ritual symbol. When users, filled with reverence and anticipation, watch the king’s boat they have personally helped build burn in the closing ritual scene, this moment is imbued with a double symbolic meaning: the various troubles carried by the boat, like heavy mist, gradually dissipate in the flames;The light turned into stardust and returned to the invisible.

### 2.3. Questionnaire design and data collection

#### 2.3.1. Cultural identification scale.

Scholars in China and abroad have developed various measurement scales around the concept of Cultural Identification. The most representative is Phinney’s Multigroup Ethnic Identity Measure (MEIM), which divides ethnic-Cultural Identification into four dimensions: ethnic self-identification, achievement of ethnic identity, ethnic behaviors, and sense of belonging [28]. Other scholars have divided consumers identification with intangible cultural heritage into cognitive, affective, and behavioral dimensions. Cognitive identity corresponds to consumers degree of familiarity with the heritage item; affective identity corresponds to the sense of belonging and closeness generated during engagement; and behavioral identity corresponds to consumers willingness to take concrete action to support the item’s transmission and development. The study integrates cognitive, affective, and behavioral identity to design a scale.

#### 2.3.2. Data collection.

This study collected data through an online questionnaire survey. Participation was entirely voluntary, and the study involved minimal risk. Ethical approval was obtained from the Ethics Committee of the Xiamen Academy of Arts and Design, Fuzhou University (Approval No. REA250825-01). Before completing the questionnaire, all participants were informed of the purpose of the study and provided online informed consent. No personally identifiable information was collected, and all data were analyzed anonymously.

A purpose sampling strategy was adopted to recruit participants from three main stakeholder groups: (1) local community members from the Fujian–Taiwan region with direct experience of the Wangchuan Ceremony; (2) intangible cultural heritage practitioners and design students familiar with digital reconstruction; and (3) members of the general public representing potential users of digital heritage experiences. Participants were recruited from September to December 2025.

The first section of the questionnaire collected participants’demographic information (Table 2), while the second section comprised 26 measurement items covering eight constructs in the research model. All items were rated on a five-point Likert scale ranging from 1 (“strongly disagree”) to 5 (“strongly agree”). A total of 240 responses were collected, of which 220 were valid and included in the analysis. To assess internal consistency, Cronbach’s alpha was calculated using IBM SPSS Statistics 27, yielding an overall α of 0.927, indicating excellent internal consistency.The questionnaire dimensions and measurement items are presented in Table 3.

**Table 2:** Demographic information.

Demographics	Characteristics	Count	Percentage (%)
Gender	Male	102	46.4
	Female	118	53.6
Age	18-25	163	74.0
	26-40	47	21.4
	41-65	8	3.6
	Above 65	2	1.0
From the Fujian and Taiwan?	Yes	139	63.2
	No	81	36.8
Understanding of ICH Rituals	Never heard of	19	8.6
	Heard of but not understood	135	61.4
	Have a certain understanding	63	28.6
	Very familiar	3	1.4
Understanding of Digital Experience Devices for ICH Rituals	Never heard of	29	13.2
	Heard of but not understood	133	60.4
	Have a certain understanding	57	25.9
	Very familiar	1	0.5

**Table 3:** Questionnaire dimensions and specific content.

Dimension	Content	References
**Perceived Design (PD)**	The device’s form design is novel	Hassenzahl, Marc [29].
	The icons, buttons, and text prompts in the device are easy to understand.	
	The color, light and shadow, and composition of the screen well reproduce the cultural atmosphere of the ICH ritual.	
	The visual design of characters, scenes, and props is very exquisite and attractive.	
	The sound effects in the device effectively set off the atmosphere and enhance my sense of immersion.	
**Behavioral Design (BD)**	The interaction method of the device is very intuitive and easy to get started.	Nielsen J [30]
	The overall experience was smooth, without interruptions or lag	
	The device provides sufficient information and guidance to let me know how to operate.	
**Emotional Design (ED)**	The narrative storyline or content in the experience can arouse my emotional resonance.	Norman, Donald [31].
	This experience makes me feel the profound heritage and value of traditional culture.	
	I think this form is more touching than just reading text or watching videos.	
**Perceived Ease of Use (PEOU)**	Learning to operate this device is easy for me.	Van der Heijden [32]; Davis, Fred [10]
	Using this device requires no excessive effort.	
	Using this device allows me to easily obtain the required cultural information.	
**Perceived Usefulness (PU)**	This experience method helps me better understand the process of the ICH ritual.	Davis, Fred [10]; Chin-Lung Hsu [33]
	It has taught me a lot of knowledge about this ICH.	
**Perceived Enjoyment (PE)**	I find the process of experience very interesting.	Davis, Fred [34]
	The experience process makes me feel happy and enjoyable.	
	The entire experience process is engaging and can attract my attention.	
**Cultural Identification (CI)**	This experience has enhanced my sense of identity and pride in this ICH.	Jean S. Phinney [28]
	Such a device experience makes me think it is very important to protect and inherit such intangible cultural heritage.	
	I feel that this ICH is related to myself to a certain extent.	
**Behavioral Intention (BI)** (Heritage-related behavioural intention: willingness to recommend, share, revisit, or further participate in and explore related intangible cultural heritage.)	I am willing to recommend digital experience projects of ICH rituals to my friends or family.	Zeithaml, Valarie [35]
	I am willing to share my experiences or feelings about the digital experience of ICH rituals on social media.	
	I will be interested in participating again if there are similar digital experiences of ICH in the future.	
	This experience has inspired my desire to personally participate in or learn more about the ICH offline.	

## Results

Data analysis was conducted using IBM SPSS Statistics 27 and SmartPLS. First, descriptive statistics and skewness and kurtosis values were examined to assess the distribution of the observed variables. Second, the measurement model was evaluated in terms of internal consistency reliability, convergent validity, and discriminant validity using Cronbach’s alpha, composite reliability (CR), average variance extracted (AVE), and factor loading. Third, partial least squares structural equation modeling (PLS-SEM) was performed to evaluate the hypothesized relationships among the constructs. Path coefficients, t-values, and p-values were examined to determine the statistical significance and strength of the hypothesized paths.

### 3.1. Normality test

Before conducting structural equation modeling (SEM), the normality of the sample data was examined to assess whether the assumption of normality was met. Bollen and Long [36] argue that the skewness and kurtosis coefficients of a measured variable’s distribution can be used to assess normality; when the absolute values of both skewness and kurtosis are below 2, the variables are generally considered to satisfy the assumption of normality. and the results are shown in Table 4.

**Table 4:** Normality test results.

Items	Indicator	Mean	Kurtosis	Skewness
**PD**	PD1	3.864	1.499	−0.691
	PD2	3.832	0.271	−0.378
	PD3	4.000	−0.459	−0.309
	PD4	4.036	−0.013	−0.416
	PD5	3.986	1.713	−0.786
**BD**	BD1	3.873	−0.242	−0.251
	BD2	3.741	−0.658	−0.167
	BD3	3.909	−0.189	−0.309
**ED**	ED1	4.005	0.396	−0.637
	ED2	4.136	0.592	−0.750
	ED3	4.173	2.322	−1.037
**PEOU**	PEOU1	3.873	0.336	−0.643
	PEOU2	3.764	−0.060	−0.449
	PEOU3	3.941	1.155	−0.682
**PU**	PU1	4.136	0.808	−0.613
	PU2	4.018	0.936	−0.760
**PE**	PE1	4.082	0.259	−0.444
	PE2	4.045	0.587	−0.71
	PE3	4.100	1.045	−0.908
**CI**	CI1	4.132	−0.459	−0.35
	CI2	4.105	−0.323	−0.437
	CI3	3.964	0.066	−0.465
**BI**	BI1	4.059	1.085	−0.738
	BI2	3.900	0.100	−0.387
	BI3	4.109	0.476	−0.698
	BI4	4.064	0.595	−0.716

### 3.2. Measurement model

Before hypothesis testing, the measurement model was assessed for reliability, discriminant validity, and convergent validity. As shown in Table 5, all factor loadings exceeded an absolute value of 0.5, indicating a significant relationship between observed variables and their latent factors; loadings ≥ 0.7 are considered excellent, indicating a sound and reliable measurement structure.

**Table 5:** Internal consistency & convergent validity.

Dimension	Factor Loading	Cronbach’s Alpha	AVE	CR
PD1	0.728	0.761	0.511	0.839
PD2	0.676			
PD3	0.729			
PD4	0.726			
PD5	0.713			
BD1	0.787	0.702	0.626	0.834
BD2	0.789			
BD3	0.798			
ED1	0.707	0.750	0.585	0.808
ED2	0.778			
ED3	0.806			
PEOU1	0.862	0.732	0.648	0.846
PEOU2	0.728			
PEOU3	0.819			
PU1	0.859	0.723	0.704	0.826
PU2	0.818			
PE1	0.852	0.766	0.682	0.865
PE2	0.790			
PE3	0.834			
CI1	0.815	0.863	0.570	0.798
CI2	0.764			
CI3	0.679			
BI1	0.797	0.800	0.626	0.870
BI2	0.713			
BI3	0.851			
BI4	0.798			

Second, Cronbach’s alpha was used to assess the internal consistency reliability of each dimension. Results showed that all dimensions had Cronbach’s alpha values between 0.702 and 0.863, indicating good internal consistency while avoiding item redundancy (Cronbach’s alpha should fall between 0.7 and 0.95 to balance reliability against redundancy).

Third, the average variance extracted (AVE) for every dimension in the model exceeded 0.5 (ranging from 0.511 to 0.798), and composite reliability (CR) ranged from 0.798 to 0.870, both meeting the criteria for convergent validity (AVE > 0.5, CR > 0.7) [37]. Taken together, the measurement model’s reliability and validity reached a good level, providing a solid basis for subsequent hypothesis testing.

Following the discriminant-validity approach proposed by Fornell and Larcker [38], when a measurement item’s standardized factor loading exceeds 0.5, this indicates that the item adequately explains its latent construct — that is, satisfactory indicator reliability [39]. In addition, the Fornell–Larcker criterion and the heterotrait–monotrait ratio (HTMT) are both established indicators of discriminant validity [40]. Under the Fornell–Larcker criterion, discriminant validity is considered acceptable if the square root of each construct’s AVE exceeds its correlations with other constructs. As shown in Table 6, the square roots of the AVEs along the diagonal meet this criterion, indicating good discriminant validity. Additionally, Henseler, Ringle, and Sarstedt recommend using the HTMT ratio to assess discriminant validity between constructs; an HTMT value below 0.9 indicates acceptable discriminant validity [41]. As shown in Table 7, all HTMT values were below 0.9, meeting this criterion and confirming the model’s good reliability and validity.

**Table 6:** Fornell–Larcker discriminant validity.

	PD	BD	ED	PEOU	PU	PE	CI	BI
**PD**	0.715							
**BD**	0.564	0.792						
**ED**	0.573	0.414	0.765					
**PEOU**	0.433	0.543	0.335	0.805				
**PU**	0.533	0.433	0.579	0.487	0.839			
**PE**	0.581	0.425	0.575	0.470	0.570	0.826		
**CI**	0.469	0.359	0.496	0.414	0.554	0.826	0.755	
**BI**	0.609	0.599	0.552	0.473	0.574	0.826	0.425	0.791

**Table 7:** HTMT ratios.

	PD	BD	ED	PEOU	PU	PE	CI	BI
**PD**								
**BD**	0.762							
**ED**	0.82	0.62						
**PEOU**	0.554	0.748	0.46					
**PU**	0.793	0.678	0.850	0.724				
**PE**	0.754	0.546	0.813	0.619	0.847			
**CI**	0.675	0.539	0.783	0.592	0.850	0.844		
**BI**	0.767	0.565	0.753	0.603	0.825	0.734	0.844	

### 3.3. Structural model

Through PLS-SEM, this study evaluates the hypothesized relationships among Perceptual Design (PD), Behavioral Design (BD), Emotional Design (ED), Perceived Ease of Use (PEOU), Perceived Usefulness (PU), Perceived Enjoyment (PE), Cultural Identification (CI) on Behavioral Intention (BI). To verify the proposed hypothetical model (see Table 8).

**Table 8:** Hypothesis testing results.

Hypotheses	Paths	Original Sample (O)	T Statistics	P Values	Supported
**H1**	PD - > PEOU	0.185	2.296	0.022	Yes
**H2**	PD - > PU	0.229	3.619	0.000	Yes
**H3**	PD - > PE	0.345	4.076	0.000	Yes
**H4**	BD - > PEOU	0.439	5.798	0.000	Yes
**H5**	BD - > PU	0.143	2.457	0.014	Yes
**H6**	BD - > PE	0.060	0.827	0.408	No
**H7**	ED - > PU	0.389	5.802	0.000	Yes
**H8**	ED - > PE	0.352	4.025	0.000	Yes
**H9**	ED - > CI	0.136	2.072	0.039	Yes
**H10**	PEOU - > CI	0.098	1.357	0.175	No
**H11**	PU - > CI	0.243	3.330	0.001	Yes
**H12**	PE - > CI	0.325	4.322	0.000	Yes
**H13**	CI - > BI	0.599	10.905	0.000	Yes

Among the external influencing factors, perceived design positively influenced users perceived ease of use, Perceived Usefulness, and Perceived Enjoyment, with significant effects on Perceived Usefulness (O = 0.229, T = 3.619, p < 0.001) and Perceived Enjoyment (O = 0.345, T = 4.076, p < 0.001), and a smaller but still significant effect on perceived ease of use (O = 0.185, T = 2.296, p < 0.050). This suggests that an aesthetically pleasing, clear visual design and multisensory experience are themselves enjoyable,that good information design directly aids understanding of cultural content, and that intuitive guidance lowers the learning threshold. Behavioral Design positively influenced perceived ease of use and Perceived Usefulness, with a significant effect on perceived ease of use (O = 0.439, T = 5.798, p < 0.001) and a comparatively weaker but still significant effect on Perceived Usefulness (O = 0.143, T = 2.457, p < 0.050), confirming that smooth, natural interaction is foundational to the experience and that fluent interaction also aids comprehension. However, Behavioral Design’s effect on Perceived Enjoyment (O = 0.060, T = 0.827, p > 0.050) was not significant — a key finding that may reveal something distinctive about digital experiences of intangible-heritage ritual: a tension exists between the solemnity of cultural experience and gamified enjoyment.Users may expect a respectful, immersive form of cultural insight rather than highly gamified form of entertainment. Emotional Design was positively associated with Perceived Usefulness, Perceived Enjoyment, and Cultural Identification, with significant effects on Perceived Usefulness (O = 0.389, T = 5.802, p < 0.001) and Perceived Enjoyment (O = 0.352, T = 4.025, p < 0.001), and a comparatively weaker but still significant effect on Cultural Identification (O = 0.136, T = 2.072, p < 0.050). This indicates that Emotional Design is highly effective at helping users understand cultural connotation, is itself engaging and enjoyable, and can bypass cognitive appraisal to directly touch users emotions.

In the cognitive-response stage, both Perceived Usefulness (O = 0.243, T = 3.330, p < 0.001) and Perceived Enjoyment (O = 0.325, T = 4.322, p < 0.001) were positively associated with cultural identification. These results suggest that both cognitive value and positive experiential responses are related to stronger Cultural Identification. This suggests, first, that when users feel the installation effectively conveys knowledge and deepens understanding, this cognitive gain translates into rational recognition and respect for cultural value;second, the experience of pleasure and enjoyment is a powerful catalyst for building Cultural Identification. When users find an experience “fun”, they will view and accept the associated culture more positively. However, perceived ease of use (O = 0.098, T = 1.357, p > 0.050) did not have a significant effect, suggesting that ease of use is a basic threshold with limited power to build a deep, affective sense of Cultural Identification.

Finally, in the affective-response stage, Cultural Identification (O = 0.599, T = 10.905, p < 0.001) showed the strongest positive association with heritage-related Behavioral Intention. This finding suggests that stronger cultural identification is associated with a greater willingness to engage in subsequent heritage-related behaviours.Within the context of heritage sustainability, this pathway represents a shift from passive consumption to active protection.when users resonate with the Wangchuan ceremony through digital interaction, their willingness to participate in and transmit the heritage is no longer driven by external novelty but stems from an internal sense of responsibility.These findings support the potential relevance of Cultural Identification (CI) as an important mechanism linking digital heritage experiences with heritage-related behavioral intention.The findings indicate that Emotional Design is positively associated with cultural identification, and cultural identification, in turn, shows a strong positive association with behavioral intention. This pattern suggests that emotionally engaging digital heritage experiences may foster stronger cultural identification, which is associated with a greater willingness to participate in heritage-related activities. Overall, these findings suggest that digital interventions may contribute to heritage-related participation when they successfully connect users experiential responses with cultural belonging.

## Discussion

### 4.1. Theoretical contributions to cultural sustainability

The study extends the Technology Acceptance Model (TAM) from the traditional field of technology adoption to the context of digital cultural heritage, offering a contribution to the growing body of research on cultural sustainability. Prior TAM research has largely focused on functional perceptions such as Perceived Usefulness and Perceived Ease Of se;the results of the study show that, in the specific context of intangible cultural heritage, technology acceptance alone is insufficient to explain why users choose to sustain their participation.

The empirical results show that cultural identification is an important factor linking user experience with behavioral intention. This finding suggests that digital heritage systems should not be understood merely as tools for information acquisition or visual representation, but also as interpretive environments that enable users to engage with cultural meanings and values. When users identify with the cultural narratives embedded in a ritual tradition, they may show greater willingness to support, share, and further explore the heritage. From the perspective of cultural sustainability, the relationship between user experience, cultural identification, and behavioral intention highlights the importance of cultural value recognition in digital heritage design. Therefore, this study extends TAM by introducing cultural identification as a cultural and sustainability-related construct, highlighting its relevance to heritage-related behavioral intention.

The findings further suggest a sequential relationship among emotional design, cultural identification, and behavioral intention. Emotional design was positively associated with perceived enjoyment and cultural identification, while perceived enjoyment was positively associated with cultural identification. Cultural identification showed the strongest positive association with behavioral intention (β = 0.599, t = 10.905, p < 0.001). These findings suggest that emotionally engaging digital heritage experiences may contribute to stronger cultural identification, which is in turn associated with greater willingness to engage with and further explore the heritage beyond the appeal of technological novelty alone. However, this sequential relationship should not be interpreted as a confirmed indirect or causal pathway, as the specific indirect effect was not directly tested in this study.Furthermore, the results suggest that digitally reconstructed rituals can function as a transformative mechanism within the heritage system.By converting embodied ritual practice into an immersive digital environment, digital reconstruction creates new forms of cultural participation that transcend the limits of time and space.In this sense, the digital ritual experience represents an emerging mode of heritage interpretation that unites technological innovation with long-term cultural sustainability.

### 4.2. Theoretical implications of the interaction design translation

The theoretical significance of this framework lies in extending the conventional “documentation–display” logic of digital heritage. It shifts the focus from what cultural elements are represented to how their cultural meanings and participatory relationships are reconstructed through interaction. In this process, ritual symbols provide the cultural semantic basis, interaction provides the experiential mechanism, and behavioural participation becomes the means through which users engage with the reconstructed ritual.It is a process of meaning-preserving transformation, in which cultural symbols are selectively interpreted, reorganized through interaction, and ultimately reconstructed as participatory experiences.

The framework also establishes a conceptual connection between intangible cultural heritage studies and interaction design by positioning interaction as an integral mechanism of cultural translation rather than a technical layer added after digitization. It emphasizes that digital heritage reconstruction should consider not only the visual representation of cultural elements but also the preservation of ritual narrative logic, symbolic relationships, and participatory functions.More broadly, this framework provides a transferable perspective for the digital transformation of ritual-based intangible cultural heritage. Although specific cultural symbols and interaction forms may vary across contexts, the proposed translation logic can provide a conceptual basis for designing and evaluating similar digital heritage experiences.

### 4.3. Policy and practical implications

The empirical findings of the study offer several actionable directions for policymakers, intangible heritage safeguarding organizations (such as UNESCO), and heritage management decision-makers.

First, a policy shift from “digital storage” to “digital empowerment.” Prior intangible heritage safeguarding policy has tended to emphasize digital archiving itself; and the findings of the study suggest that cultural identification is the key intermediary for achieving sustainable inheritance.Policymakers might therefore consider providing greater support for people-centered digital transformation projects by prioritizing interactive designs that foster emotional engagement and encourage public participation, rather than focusing solely on high-precision 3D scanning.

Second, the incentive mechanism for low-carbon heritage protection. In response to the environmental burden brought about by the “burning the Wangchuan boat” (烧王船), government departments can explore incentive policies in terms of green non-heritage: by promoting the “digital-entity integration” model proposed in the study, communities are encouraged to replace some highly polluting physical consumption links with digital means.This approach both echos with the climate-action goals of the Sustainable Development Goals (SDG 13) and offers a viable path for the continuation of folk activities under increasingly restrictive urban environmental conditions.

Third, the community and decentralization of cultural governance. Digital transformation should empower communities rather than being driven solely by government. It is recommended that UNESCO establish public-participation evaluation indicators based on digital interaction.The TAM + CI framework validated in this study could be used to quantify the social sustainability of intangible-heritage projects, enabling more scientifically informed allocation of international protection resources.

## Limitations and future research

There are some limitations in the study. First of all, the representative and geographical coverage of the sample are still limited. Although 220 participants provide a reliable baseline for structural equation model (SEM) analysis, the sample is mainly composed of young individuals with digital literacy.While this group represents a key target population for heritage transformation, it means that this study’s conclusions regarding “cultural identification” and “inter-generational transmission” should be understood as applying primarily to the digital-native generation’s acceptance of this type of digital experience, and cannot be directly generalized to older practitioners or to communities outside the Fujian–Taiwan region, which is precisely the groups that constitute the core of the “transmission gap” repeatedly referenced in this paper.Future research should incorporate a broader range of local older community members and practitioners who hold traditional ritual knowledge, and should extend the study’s geographic scope to other communities along the Maritime Silk Road, such as Vietnam and Thailand, to further validate the generalization of this framework.

Secondly,

Second, no control group was included in this study. All participants viewed the same concept video and completed the same questionnaire, so it was not possible to compare the proposed design against alternative conditions, such as text-based information, a non-emotional design version, or no intervention. As a result, the observed associations may be influenced by novelty effects, social desirability, or other unmeasured factors. Future research should use randomized controlled designs or multi-group comparisons to isolate the specific contribution of the digital interaction design.

Third, the proposed digital interaction system was not directly tested with participants. The questionnaire examined participants existing experiences and perceptions of digital cultural experiences and intangible cultural heritage rather than their actual use of the proposed interaction system. Therefore, the findings cannot establish the actual usability, emotional engagement, or experiential effectiveness of the proposed system. Future research should conduct prototype-based user testing to further evaluate the usability and experiential outcomes of the proposed interaction design.

Finally, although the structural model identified significant associations among emotional design, perceived enjoyment, cultural identification, and behavioral intention, the specific indirect effect of the full sequential relationship was not directly tested. Therefore, the relationship among emotional design, perceived enjoyment, cultural identification, and behavioral intention should be interpreted as a theoretically supported sequential association rather than a confirmed serial mediation effect. Future research should employ formal mediation analysis to further examine the indirect relationships among these constructs.

## Conclusion

Using the Wangchuan ceremony — a maritime intangible cultural heritage shared by China and Malaysia — as a case study, this research provides empirical evidence on the digital transformation of intangible cultural heritage. By integrating an extended Technology Acceptance Model (TAM), emotionally resonant design, and cultural identification (CI), this study examines the relationships among users digital heritage experiences, cultural identification, and heritage-related behavioural intention.

Based on the above analysis, this study reaches three main conclusions. First, the “digital–physical hybrid” model is not simply a technological substitute but provides a potential approach for balancing traditional ritual practice with the demands of the modern environment. By creating immersive and emotionally engaging experiences, digital intervention may reduce reliance on some physical consumption while maintaining key cultural meanings and social functions of rituals.

Second, the findings highlight the important role of cultural identification (CI) in heritage-related behavioural intention. Cultural identification showed the strongest positive association with behavioural intention (β = 0.599, p < 0.001), while emotional design was positively associated with perceived enjoyment (β = 0.352, p < 0.001), and perceived enjoyment was positively associated with cultural identification (β = 0.325, p < 0.001). Taken together, these findings suggest a sequential relationship in which emotionally engaging digital heritage experiences may contribute to stronger cultural identification, which is in turn associated with greater willingness to engage with and further explore the heritage. However, the full sequential indirect effect was not directly tested in this study and should therefore not be interpreted as a confirmed serial mediation effect. These findings suggest that future digital heritage projects should pay greater attention to reflective and emotional participation alongside technological innovation.

Third, the digital transformation of the Wangchuan ceremony provides a case-based reference for exploring digital heritage practices along the Maritime Silk Road and its surrounding regions. By connecting local cultural knowledge with broader sustainable development goals, digital innovation may help broaden access to heritage and support cross-community cultural exchange. The shift from static archival preservation toward interactive digital experiences represents a potential direction for the continued exploration of digital approaches to cultural heritage protection and transmission.

## Supporting information

S1 Video Demonstration of the proposed digital Wangchuan ceremony installation. A design demonstration illustrating the proposed installation and its intended interactive experience. Created by the authors.
(MP4)
